# The Disordered *C*-Terminus of the Chaperone DnaK Increases the Competitive Fitness of *Pseudomonas putida* and Facilitates the Toxicity of GraT

**DOI:** 10.3390/microorganisms9020375

**Published:** 2021-02-13

**Authors:** Sirli Rosendahl, Andres Ainelo, Rita Hõrak

**Affiliations:** Department of Genetics, Institute of Molecular and Cell Biology, University of Tartu, Riia St 23, 51010 Tartu, Estonia; sirliluup@gmail.com (S.R.); andres.ainelo@gmail.com (A.A.)

**Keywords:** toxin-antitoxin system, GraTA of HigBA family, DnaK chaperone, disordered domain, competitive fitness, *Pseudomonas putida*

## Abstract

Chaperone proteins are crucial for proper protein folding and quality control, especially when cells encounter stress caused by non-optimal temperatures. DnaK is one of such essential chaperones in bacteria. Although DnaK has been well characterized, the function of its intrinsically disordered *C*-terminus has remained enigmatic as the deletion of this region has been shown to either enhance or reduce its protein folding ability. We have shown previously that DnaK interacts with toxin GraT of the GraTA toxin-antitoxin system in *Pseudomonas putida*. Interestingly, the *C*-terminal truncation of DnaK was shown to alleviate GraT-caused growth defects. Here, we aim to clarify the importance of DnaK in GraT activity. We show that DnaK increases GraT toxicity, and particularly important is the negatively charged motif in the DnaK *C*-terminus. Given that GraT has an intrinsically disordered *N*-terminus, the assistance of DnaK is probably needed for re-modelling the toxin structure. We also demonstrate that the DnaK *C*-terminal negatively charged motif contributes to the competitive fitness of *P. putida* at both high and optimal growth temperatures. Thus, our data suggest that the disordered *C*-terminal end of DnaK enhances the chaperone functionality.

## 1. Introduction

Proper folding is of utmost importance for proteins to be able to complete their biological function. While most proteins have the intrinsic ability to obtain their correct structure, the folding process is essentially promoted by cellular chaperones. Chaperones not only assist the folding of newly synthesized proteins, but they also contribute to protein quality control by resolving and refolding misfolded protein aggregates or by delivering the aberrant proteins to cellular proteases for degradation [1,2]. Chaperones become particularly important under stress conditions, such as low or high temperature, heavy metals and oxidative stress, that promote protein misfolding and aggregation [3,4].

DnaK, with its co-chaperones DnaJ and GrpE, comprises an important component of the protein quality control chaperone network in bacteria [5,6]. DnaK is composed of an *N-*terminal nucleotide-binding domain (NBD), a *C*-terminal substrate-binding domain (SBD) and a short intrinsically disordered *C*-terminal tail with unclear function [7,8,9]. ATP binding and hydrolysis by DnaK NBD allosterically controls the binding of SBD to its substrates—short hydrophobic peptide segments that would normally be buried in the folded structure. This binding in turn enhances ATP hydrolysis by NBD [10,11]. DnaK facilitates the folding through repeated cycles of ATP-dependent binding and release of an unfolded protein [6]. While the *C*-terminal conserved disordered tail is not necessary for DnaK chaperone activity [12,13,14], it has been hypothesized to serve as an additional binding site for unfolded proteins [7]. However, the physiological importance of this possible auxiliary interaction between DnaK and its substrate proteins has remained controversial, as it is unclear whether the truncation of the *C*-terminal tail enhances [8] or reduces the protein refolding activity of DnaK [7].

Interestingly, folding-assisting chaperones have been shown to be associated with the regulation of some toxin-antitoxin (TA) systems [15,16,17]. TA systems represent a family of two-gene loci that are widespread in mobile DNA, such as plasmids, transposons and phages, but also in bacterial chromosomes [18,19]. TA systems encode a toxic protein able to damage crucial biological processes or structures [20], and its antagonist that holds the toxin under strict control [21]. TA loci located in mobile elements serve as addiction modules that ensure stable maintenance of the mobile DNA [22,23]. This is conferred by the different stability of the toxin and the antitoxin. As antitoxins are usually less stable than toxins, the loss of mobile DNA results in the release of toxins, and thus growth suppression or death of the host [24,25]. Structural analysis on TA proteins has revealed that many antitoxins contain an unstructured region, which makes them susceptible to proteolysis [26,27]. Interestingly, some TA systems have recruited a chaperone gene to modulate the antitoxin stability. For example, the activity of a tripartite toxin-antitoxin-chaperone (TAC) module in *Mycobacterium tuberculosis* is controlled by a SecB-like chaperone [15,28]. The SecB homolog of the TAC operon contributes to toxin neutralization as the chaperone stabilizes the otherwise unstable antitoxin [15,16]. DnaK could be involved in the regulation of TA systems as well, as several *E. coli* antitoxins, i.e., DinJ, MazE, RelB and MqsA, belong to the DnaK interactome [5]. Intriguingly, in the *Pseudomonas putida* GraTA system, instead of the antitoxin, the toxin GraT was shown to interact with DnaK, and mutations in *dnaK* suppress GraT toxicity [17].

GraT is a member of the HigB family of toxins that belong to a RelE/ParE superfamily, which commonly encode ribosome-dependent RNases. In accordance with that, GraT is a ribosome-dependent mRNase toxin [29] which is neutralized by the antitoxin GraA [30]. The toxicity of GraT is highly temperature-dependent, being negligible at higher temperatures but gradually increasing at lower temperatures [30]. Atypical to an mRNase toxin, GraT causes a specific cold-dependent ribosome biogenesis defect characterized by the accumulation of ribosome precursors stalled at the late stages of their maturation [17]. The host bacterium tries to alleviate this GraT-caused defect by the upregulation of ribosome biogenesis factors, as evidenced by proteome analysis of the antitoxin deletion strain *ΔgraA* [31]. GraT and GraA differ structurally from most other TA proteins as not the antitoxin but the toxin possesses an intrinsically disordered region [29]. While most antitoxins contain unstructured segments which make them susceptible to proteases [27,32], the antitoxin GraA is fully folded [29]. Consistently, GraA is an uncommonly stable protein, though its stability is decreased at the onset of stationary phase [33]. Unlike its closest homolog HigB and other TA toxins that are fully folded proteins [27,34], the toxin GraT contains an intrinsically disordered *N*-terminus [29]. This unstructured region of the toxin plays a dual role in GraTA regulation: it is required for GraT mRNase activity and toxicity, and it also acts as a derepressor of the *graTA* operon by preventing the binding of the GraTA complex to the operator [29]. Considering the partial disorder of GraT, it is highly interesting that GraT can interact with DnaK and that the *C*-terminal truncation of DnaK alleviates the GraT-caused ribosome biogenesis defect [17]. While the importance of the GraT-DnaK interaction remained unclear, two alternative hypotheses were proposed to explain the involvement of DnaK in the GraT-caused ribosome biogenesis defect [17]. The first hypothesis relies on the fact that DnaK participates in ribosome biogenesis [35,36], and poses that GraT directly attacks DnaK and inhibits its ability to assist ribosome maturation. The alternative hypothesis is that DnaK supports GraT toxicity, possibly by re-modelling the GraT structure.

Here, we aimed to clarify the role of DnaK in GraT toxicity. Our data suggest that DnaK enhances the GraT-caused phenotypes and that the most distal *C*-terminal end of DnaK is particularly important in the ability of DnaK to enhance GraT toxicity. We also addressed the question about the importance of the DnaK *C*-terminal disordered tail in *P. putida* fitness.

## 2. Materials and Methods

### 2.1. Bacterial Strains, Plasmids, and Growth Conditions

The bacterial strains and plasmids are listed in Table 1. *P. putida* strains used are derivatives of PaW85 [37], which is isogenic to the strain KT2440 [38,39]. Bacteria were grown in lysogeny broth (LB) medium. If selection was necessary, the growth medium was supplemented with ampicillin (100 µg mL^−1^) or kanamycin (50 µg mL^−1^) for *E. coli* and benzylpenicillin (1500 µg mL^−1^), kanamycin (50 µg mL^−1^) or streptomycin (200 µg mL^−1^) for *P. putida*. Unless noted otherwise, *E. coli* was incubated at 37 °C and *P. putida* at 30 °C. Electrotransformation was carried out as described in [40].

### 2.2. Construction of Plasmids and Strains

Oligonucleotides used in PCR amplifications are listed in Table 2. For the creation of DnaK overexpression strains, the *lacI^q^-P_tac_-dnaK* expression cassette was constructed. The *dnaK* gene fragment was amplified from the *P. putida* PaW85 chromosome with primers DnaKBam and dnaJdel, cut with EcoRI and BamHI and cloned into EcoRI-BamHI-opened pUCNot-lacItac. The *lacI^q^-P_tac_-dnaK* cassette was subcloned as a NotI fragment into the miniTn7 delivery plasmid pBK-miniTn7-ΩSm. The obtained pminiTn7lacItac-dnaK was introduced into *P. putida* wild-type and *ΔgraA* strains by co-electroporation together with the helper plasmid pUXBF13. The presence of the expression cassette in the *attTn7* site of the tac-*dnaK* and *ΔgraA* tac-*dnaK* strains was verified by PCR.

For replacing the genomic wild-type *dnaK* allele with the *dnaK_mut4_*, site-directed mutagenesis of *dnaK* was performed. For that, the upstream and downstream regions of *dnaK C*-terminus to be mutated were amplified separately with primer pairs dnaKKpn/dnaK-3E1D and K-3E/dnaJXba, respectively. Two PCR products were joined into an approximately 1-kb fragment by overlap extension PCR using primer pair dnaKKpn/dnaJXba. The PCR fragment was digested with KpnI and XbaI and cloned into pEMG. The obtained pEMG-dnaK_mut4_ plasmid was delivered into *P. putida* PaW85 or *ΔgraA* by electroporation, and after 3 h of growth in LB medium the bacteria were plated onto LB agar supplemented with kanamycin. Kanamycin-resistant cointegrates were selected and electrotransformed with the I-SceI expression plasmid pSW(I-SceI). To resolve the cointegrate, the plasmid-encoded I-SceI was induced with 1.5 mM 3-methylbenzoate overnight. Kanamycin-sensitive colonies were selected and the deletions were verified with PCR. The plasmid pSW(I-SceI) was eliminated from the deletion strains by growing them overnight in LB medium without antibiotics.

For the construction of *secB* deficient strains, the *secB* gene was amplified from the *P. putida* PaW85 genome with primers secBalg and secBlopp and cloned into XhoI-XbaI-opened pBluescript KS. In the obtained pKS-secB plasmid, the central 280-bp Van91I-PstI sequence of *secB* was replaced with the Sm^r^ gene, and the resulting *secB*::Sm sequence was subcloned as a SacI-Acc65I fragment into pGP704L. The interrupted *secB* gene was inserted into the chromosome of *P. putida* PaW85 and the *dnaK_mut4_* strains by homologous recombination. Plasmid p704L/secB::Sm was conjugatively transferred from *E. coli* CC118 λ*pir* into *P. putida* using the helper plasmid pRK2013. The *secB* deficiency of the strain was verified by PCR analysis.

For the construction of *dnaK_mut4_*Sm and *dnaK_mut4_*Km strains, miniTn7 delivery plasmids pBK-miniTn7-ΩSm or pBK-miniTn7-Km, respectively, together with pUXBF13 helper plasmid were coelectroporated into *dnaK_mut4_*. Streptomycin or kanamycin resistant bacteria were selected and the miniTn7 insertion to *glmS* locus was verified by PCR.

The plasmids for the Bacterial Two-Hybrid (BACTH) assay were constructed as follows. The *graT* gene was amplified with primer pair T25-graT/1586Bam, and the PCR product was cleaved with PstI and BamHI and inserted into corresponding sites in pT25 to yield plasmid pT25-graT. Plasmid pT18-graA was constructed by the amplification of *graA* gene using primer pair T18-graA/1585Bam and cloning of the SalI-BamHI-cleaved PCR product into pT18. For the construction of plasmid pT18-dnaK_C44_, the end of *dnaK* gene was amplified by primers T18-dnaKsaba and dnaJdel and the KpnI-EcoRI-cleaved PCR product was cloned into corresponding sites in pT18.

In order to construct the protein expression plasmid pET-hisDnaK_C57_ for the pull-down assay, the *C*-terminal 57 amino acids of DnaK were fused with *N-*terminal His_6_ tag by PCR with the aid of oligonucleotides his-dnaK(C57) and dnaKSmaBam. The resulting PCR fragment was treated with NdeI and BamHI and inserted into the corresponding sites in the pET11c plasmid.

### 2.3. qRT-PCR

Total RNA for *dnaK* mRNA quantification was isolated from exponential-phase bacteria using the RNAzol^®^ RT and BAN (4-bromoanisole) reagents (Molecular Research Center, Inc., Cincinnati, OH, USA). The qRT-PCR assay was performed on the Rotor-Gene Q system (QIAGEN, Hilden, Germany) using the SuperScript^®^ III Platinum^®^ SYBR^®^ Green One-Step qRT-PCR Kit (Thermo Fisher Scientific, Waltham, MA, USA) according to the manufacturer’s protocol, except with double primer concentrations. For each reaction, 1 ng of total RNA was used. The *dnaK* gene was amplified using primers dnaKqkeskFw and dnaKqkeskRev, and the *rpoD* gene was amplified using primers rpoDqFw and rpoDqRev. Raw data were analyzed with the Rotor-Gene Q software v. 2.02 (QIAGEN) and mRNA amounts were calculated using the LinRegPCR software v. 2013.0 [51]. Data from at least three independent qRT-PCR experiments performed on two independently extracted RNAs were averaged and normalized against *rpoD* levels.

### 2.4. Temperature and Stress Tolerance Assays

After the bacteria were grown overnight in 5 mL of LB medium, 10-fold serial dilutions of the cultures were spotted as 5 μL drops onto LB plates with or without added chemicals (specified in Results). Plates were incubated at indicated temperatures for 24 or 48 h.

### 2.5. Growth Curves

The optical density of overnight bacterial cultures at 580 nm was measured and the cells were diluted in fresh LB medium for OD_580_ to be 0.1. Aliquots of 100 µL were transferred into microtiter plate wells and the cells were grown with shaking at 750 rpm at 30 °C, 34 °C or 37 °C, and OD_580_ was measured every 30 min with a TECAN Sunrise^TM^ microplate reader.

### 2.6. Pull-Down Assay

The assay was performed as described previously [17]. Briefly, *E. coli* BL21(DE3) cultures carrying the pET-hisDnaK, pET-hisDnaK_C57_, pET-graT_Δ1C_ or pET-Δ22graT plasmids were pre-grown overnight at 37 °C and diluted into 200 mL fresh LB medium to a starting OD_580_ of around 0.1. All bacteria were grown at 30 °C for 2 h, after which the cultures expressing GraT_Δ1C_ and Δ22GraT were transferred to 25 °C, while those expressing the DnaK variants remained at 30 °C. Protein expression was induced by the addition of 0.5 mM IPTG (Isopropyl β-d-1-thiogalactopyranoside) at an OD_580_~0.6. After ~5 h of induction, the cells were pelleted and stored at −20 °C. For protein binding and purification, two pellets containing the proteins of interest were resuspended (50 mM Tris pH 8.5; 0.5 M NaCl; 10 mM imidazole), mixed together and sonicated. The lysate was cleared by centrifugation and filtered through a 0.22 μm filter before loading it onto a 1 mL HisTrap HP (Cytiva, Uppsala, Sweden) column equilibrated with buffer A (50 mM Tris pH 8.5; 0.5 M NaCl; 50 mM imidazole). Protein purification was performed by fast protein liquid chromatography (FPLC) using an Äkta Prime chromatography system (GE Healthcare Life Sciences). Proteins were eluted using a linear 50–600 mM imidazole gradient. Samples from elution fractions with peak absorbance at 280 nm were collected, denatured and separated on 10% Tricine-SDS-PAGE. In parallel with Coomassie staining, the same samples were subjected to Western blotting using anti-GraT mouse antisera and alkaline phosphatase-conjugated goat anti-mouse antibodies. The blots were developed using bromochloroindolyl phosphate/nitro blue tetrazolium chloride (BCIP/NBT).

### 2.7. Bacterial Two-Hybrid Assay (BACTH)

The adenylate cyclase deficient *E. coli* reporter strain BTH101 was co-electroporated with two recombinant plasmids encoding hybrid proteins in which the proteins of interest were fused with either T25 or T18, i.e., fragments of *Bordetella pertussis* adenylate cyclase [46]. β-galactosidase activity was measured from bacteria grown on LB solid medium supplemented with 1 mM IPTG.

### 2.8. Measurement of Heat Shock Response

*P. putida* carrying the reporter plasmid pAG032 was grown at 30 °C in glucose minimal medium until OD_600_~0.5. Then, some cultures were shifted to either 34 °C or 38 °C and after two hours of the temperature shift the OD_600_ and fluorescence (excitation 433 nm; emission 475 nm) were measured in a TECAN Infinite M200 PRO microplate reader. Fluorescence values were normalized to the OD_600_.

### 2.9. Competition Assay

*P. putida* wild-type and *dnaK_mut4_* strains that are marked with an antibiotic resistance gene (streptomycin or kanamycin) were grown overnight in 5 mL LB medium at 30 °C. The optical densities of the bacterial cultures at 580 nm were measured and mixtures containing equal amounts of wtSm and *dnaK_mut4_*Km or wtKm and *dnaK_mut4_*Sm cells were prepared. The 1:1 mixtures were diluted 10,000-fold (about 10^5^ cells per ml) into fresh 5 mL of LB and grown at 30 °C or 34 °C. Cells were diluted into fresh LB medium every 2 days and CFU/mL was measured every 4 days.

## 3. Results

### 3.1. DnaK mRNA Levels Are Unaltered in the ΔgraA Strain

**Figure 1:**
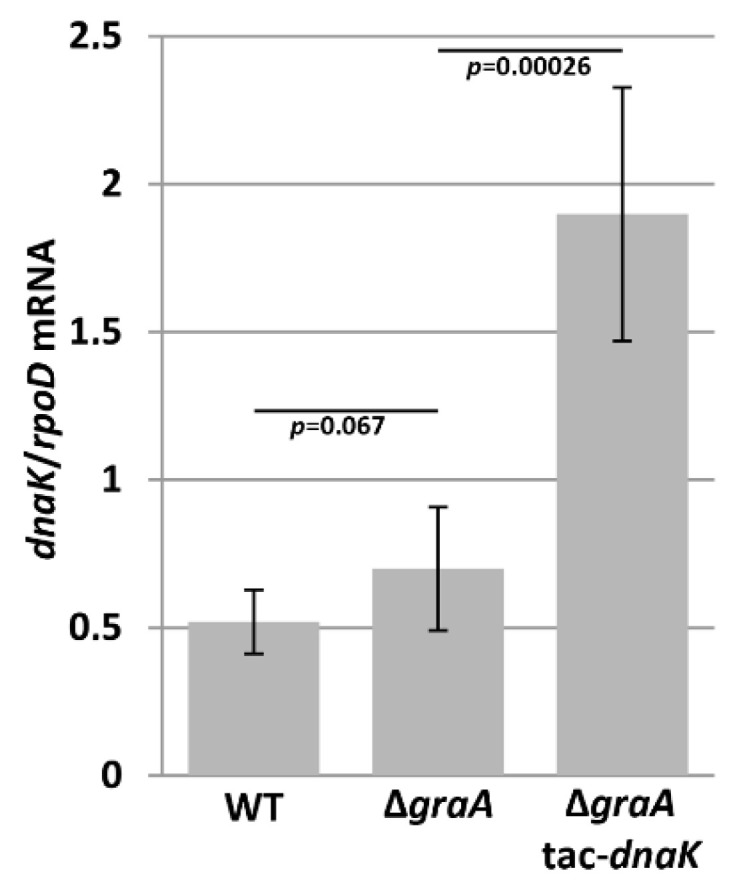
The *dnaK* mRNA levels are not affected by the GraT toxin. The relative abundance of the *dnaK*-specific mRNA in *P. putida* wild-type PaW85 (WT), Δ*graA* and Δ*graA* tac-*dnaK* strains were determined by qRT-PCR with *rpoD* as a reference gene. For induction of DnaK from the tac-*dnaK* cassette, 0.5 mM IPTG was used. Error bars represent the 95% confidence intervals from at least three independent experiments. *p* values of Student’s *t*-test are indicated.

Our previous data suggest that the GraT-caused growth inhibition and ribosome biogenesis defect is somehow associated with the chaperone and ribosome assembly factor DnaK [17]. Interestingly, whole-cell proteome comparison revealed that DnaK levels tended to be decreased in the Δ*graA* strain compared to wild-type, although the change was not statistically significant [31]. Therefore, considering that GraT is a ribosome-dependent mRNase [29], we hypothesized that GraT may target DnaK mRNA and the decreased abundance of DnaK results in the ribosome biogenesis defect. To test this possibility, we measured the *dnaK* mRNA levels in the *P. putida* wild-type and the Δ*graA* strain grown at 25 °C, where the GraT induces remarkable growth retardation [30]. qRT-PCR analysis revealed no significant differences in *dnaK* mRNA abundances between the wild-type and Δ*graA* strains (Figure 1). This refutes the possibility of *dnaK* mRNA being a specific target of GraT, and thus that the ribosome biogenesis defect may stem from DnaK depletion.

### 3.2. DnaK Enhances the Toxicity of GraT

**Figure 2:**
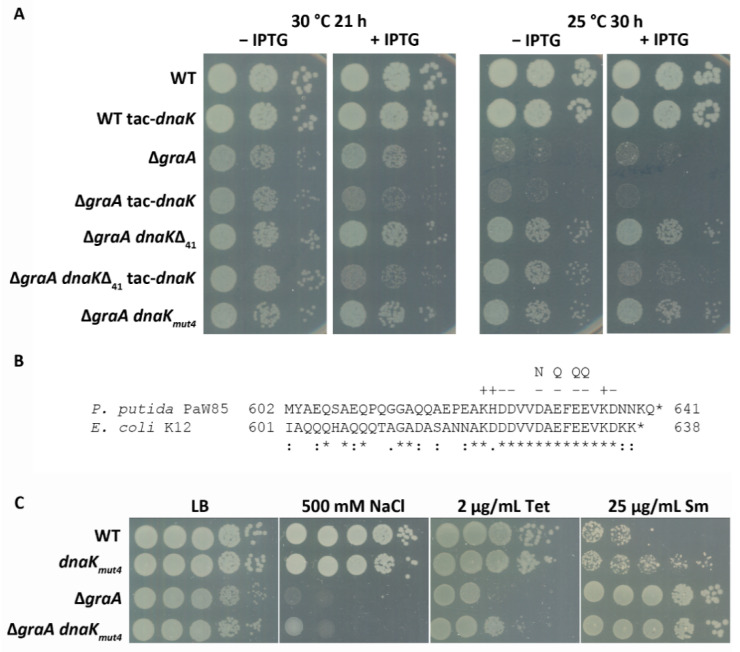
The conserved *C*-terminal domain of the DnaK is important for GraT toxicity. (**A**) Growth assay on a solid medium of the *P. putida* wild-type PaW85 (WT), Δ*graA* and their derivatives encoding the tac-*dnaK* overexpression cassette as well as Δ*graA dnaK*_Δ41_ and Δ*graA dnaK_mut4_* strains encoding the DnaK with truncated or mutated *C*-terminus, respectively. Tenfold dilutions were spotted onto lysogeny broth (LB) medium and LB supplemented with 0.5 mM IPTG. Agar plates were incubated at 30 °C or 25 °C for indicated times. (**B**) Sequence alignment of *P. putida* PaW85 and *E. coli* K12 DnaK chaperone *C*-terminal ends. The charged amino acids in the conserved *C*-terminal motif are marked with “−“ and “+”. The four substitution mutations (D629N, E631Q, E633Q and E634Q) in the motif are indicated by N and Q. (**C**) Solid medium stress tolerance assays of *P. putida* wild-type, Δ*graA* and their *dnaK_mut4_* derivatives. Tenfold serial dilutions of the overnight cultures were spotted onto LB medium and on LB supplemented with NaCl, tetracycline or streptomycin. Final concentrations of the chemicals are indicated. Plates were incubated at 30 °C for 24 h (LB) or 48 h.

GraT is a structurally exceptional protein among TA toxins as it contains an intrinsically disordered region, which is important for its activity [29]. Considering this and the finding that GraT can bind DnaK [17], we hypothesized that DnaK may assist GraT folding and enhance its toxicity. Notably, when searching for GraT suppressors, we identified only specific *dnaK* mutants where the transposon had disrupted the most distal *C*-terminal end of DnaK [17]. To further test whether DnaK is needed for GraT toxicity, we tried to construct the Δ*dnaK* and Δ*graA*Δ*dnaK* strains, but this turned out to be impossible, indicating that DnaK is essential for *P. putida*. We therefore used another approach and analyzed whether the chaperone overexpression could enhance the GraT effects. Thus, we introduced an inducible copy of *dnaK* into the Δ*graA* chromosome and evaluated the cold tolerance of bacteria. DnaK overexpression was verified by qRT-PCR at 25 °C, where 2.7-fold more *dnaK* mRNA was detected in the Δ*graA*-tac-*dnaK* strain compared to Δ*graA* (Figure 1). Growth assay on solid medium indicates that DnaK overexpression slightly increases the growth defect of Δ*graA* both at 30 °C and 25 °C (Figure 2A). As a control, we confirmed that the induction of DnaK in wild-type background has no effect on bacterial growth (Figure 2A). To further validate the model where DnaK enhances the GraT-caused growth inhibition, we also overexpressed DnaK in a Δ*graA dnaK_Δ41_* strain. This strain encodes for a truncated DnaK_Δ41_ (lacking the 41 *C*-terminal amino acids) that largely suppresses GraT effects [17]; (Figure 2A). The overexpression of DnaK in the Δ*graA dnaK_Δ41_* strain overrides the alleviating effect of the *dnaK_Δ41_* allele and restores the cold-sensitive phenotype (Figure 2A). This result not only supports the positive role of DnaK in the GraT-caused growth defect, but also suggests that the *C*-terminal end of the chaperone plays a particularly important role in promoting GraT toxicity.

### 3.3. A Negatively Charged Motif in the DnaK C-Terminus Is Important for GraT Toxicity

The extreme *C*-terminus of DnaK is highly conserved (Figure 2B), and it has been proposed that this sequence acts as an auxiliary binding site for denatured proteins [7]. In order to test whether a specific feature of this conserved motif is important for supporting GraT toxicity, site-directed mutagenesis of DnaK was performed. Four negatively charged amino acids (D629, E631, E633 and E634) in the conserved end of DnaK were substituted with the respective polar residues Asn or Gln (Figure 2B). The wild-type *dnaK* gene in the Δ*graA* genome was replaced with the mutated *dnaK_mut4_* allele, and bacterial growth at 25 °C was analyzed. Data show that, similar to the *C*-terminus deletion, substitutions within the conserved end of DnaK significantly alleviate the growth defect of Δ*graA* (Figure 2A). Given that GraT has opposite effects on the stress tolerance of the Δ*graA* strain [30], we also analyzed whether the GraT-caused tolerance effects can be reversed or alleviated if the DnaK *C*-terminus is mutated. For that, we compared the stress tolerance of wild-type, *dnaK_mut4_*, Δ*graA*, and Δ*graA dnaK_mut4_* strains on solid media supplemented with streptomycin, tetracycline or excess of NaCl. As expected, the tolerance of the Δ*graA* strain to tetracycline and NaCl was increased if this strain carried the *dnaK_mut4_* allele (Figure 2C). This is consistent with the finding that the *C*-terminal negatively charged motif contributes to the ability of DnaK to support GraT toxicity. Surprisingly, the tolerance of the Δ*graA* strain to streptomycin was not affected by the mutated *dnaK* gene (Figure 2C). However, we observed that the *dnaK_mut4_* strain displayed slightly higher streptomycin tolerance than the wild-type *P. putida* (Figure 2C). Thus, the mutated DnaK seems to affect streptomycin tolerance independent of its effects on GraT activity.

By using an in vitro pull-down assay, we have previously demonstrated that GraT binds to DnaK and also to its *C*-terminal truncation derivatives DnaK_Δ41_ and DnaK_Δ66_, which have retained their substrate binding domain [17]. However, as DnaK_Δ41_ and DnaK_Δ66_ are less efficient chaperones for GraT, the robust binding of the toxin to the SBD of DnaK seems to be insufficient to achieve full activation of GraT. To test whether the *C*-terminal domain of DnaK can give additional interactions with GraT, we performed an in vitro pull-down assay as well as used the bacterial two-hybrid (BACTH) system to detect protein binding in vivo.

**Figure 3:**
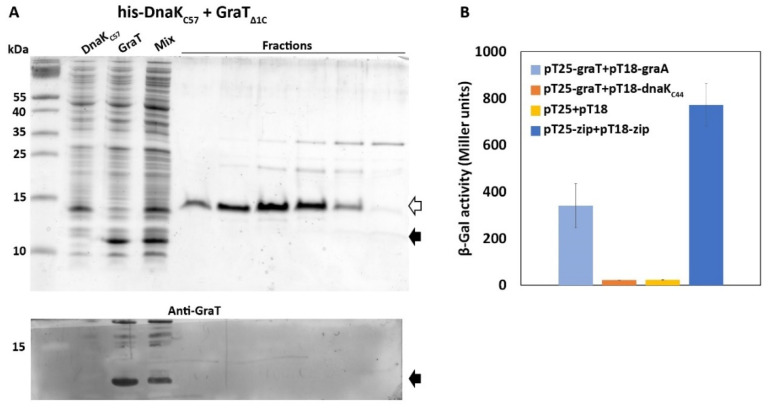
Stable interaction between GraT and *C*-terminal domain of DnaK could be detected neither in vitro nor in vivo. (**A**) An in vitro pull-down assay with His_6_-DnaK_C57_ as a bait protein for GraT_Δ1C_. The cell lysate (Mix) prepared by mixing two *E. coli* cultures overexpressing either His_6_-DnaK_C57_ or GraT_Δ1C_ was loaded onto Ni^2+^ affinity column and column elution fractions were analyzed on Coomassie-stained SDS-PAGE gel. Lower panel represents the Western blot analysis of an identical gel with anti-GraT antibodies. His_6_-DnaK_C57_ and GraT_Δ1C_ are indicated by the empty and solid arrows, respectively. (**B**) An in vivo bacterial two-hybrid assay for analyzing the functional complementation between T25 and T18 fragments of *Bordetella pertussis* CyaA. β-galactosidase activities were measured in *E. coli* BTH101(Δ*cyaA*) co-transformed with two plasmids that encode proteins of interest (GraT, GraA, and 44 amino acid-long *C*-terminal end of DnaK) fused with T25 and T18. Plasmid pairs pT25+pT18 and pT25-zip+pT18-zip served as negative and positive controls, respectively. Bacteria were grown overnight at 37 °C on LB agar plates supplemented with 1 mM IPTG. The results are the average of four independent replicates and error bars represent the standard deviation.

For the pull-down assay, *E. coli* expressing His_6_-DnaK_C57_ (the *C*-terminal 57 amino acids of DnaK were fused with *N-*terminal His_6_ tag) was mixed with cells expressing the nontoxic GraT_Δ1C_ (lacks *C*-terminal histidine residue), and the mixed cell lysate was run through a Ni^2+^ affinity column. Analysis of column eluates on SDS-PAGE revealed purification of the His_6_-DnaK_C57_, but not the GraT_Δ1C_ (Figure 3A). This result is consistent with previous data reporting that glutathione *S*-transferase fusion to the DnaK *C-*terminal end did not pull down any proteins from *E. coli* lysates [7]. Still, we tested whether the possible interaction between DnaK *C*-terminus and GraT can be detected in an in vivo BACTH assay [46]. For that, GraT was fused with the T25 fragment of the CyaA (plasmid pKT25-graT), and the 44 *C*-terminal amino acids of DnaK were fused with the T18 fragment of CyaA (plasmid pUT18C-dnaK_C44_). As GraT binds its antitoxin GraA, GraA was also fused with the T18 fragment (plasmid pUT18C-graA) as a positive control. As expected, the co-expression of T25-graT and T18-graA fusions in *E. coli* BTH101 resulted in the activation of the *lacZ* reporter (Figure 3B). However, no reporter activity exceeding the negative control was detected when T25-graT was co-expressed with T18-dnaK_C44_ fusion (Figure 3B). Thus, neither pull-down nor BACTH assays give evidence of a stable interaction between GraT and the *C*-terminus of DnaK.

### 3.4. The Disordered N-Terminus of GraT Is Not Essential for Binding to DnaK

**Figure 4:**
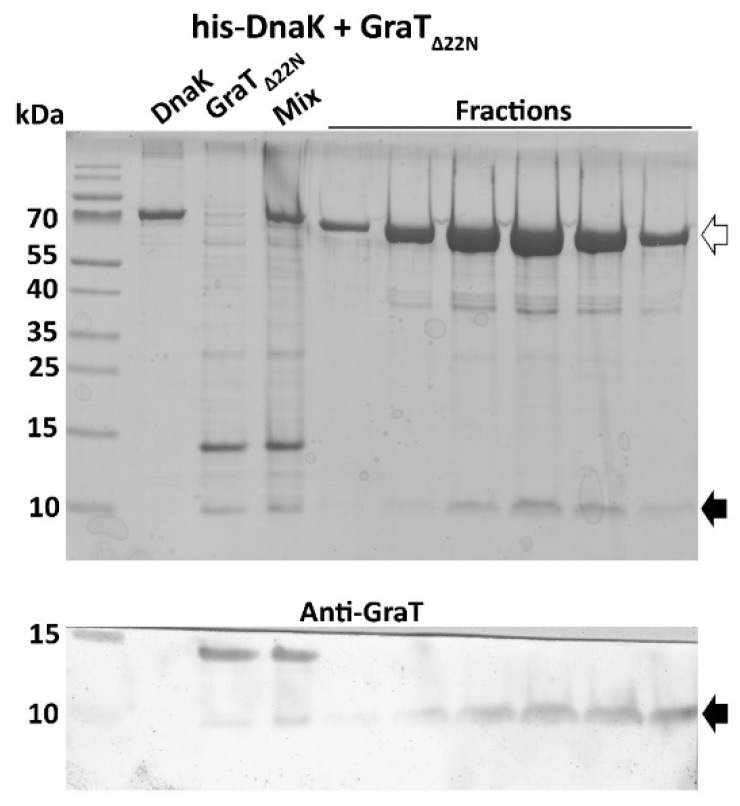
DnaK binds to *N*-terminally truncated GraT. His_6_-DnaK pulls down the *N*-terminally truncated toxin GraT_Δ22N_ from the cell lysate (Mix) prepared by mixing two *E. coli* cultures overexpressing either His_6_-DnaK (DnaK) or GraT_Δ22N_. Upper panel shows the Coomassie-stained lysates (DnaK, GraT_Δ22N_, Mix) and elution fractions from the Ni^2+^ affinity column resolved by SDS-PAGE. Lower panel represents the Western blot analysis of an identical gel with anti-GraT antibodies. His_6_-DnaK and GraT_Δ22N_ are indicated by the empty and solid arrows, respectively.

GraT is a structurally unusual protein among TA-encoded toxins as it contains an intrinsically disordered *N*-terminus [29]. Given that DnaK interacts with unstructured proteins, we hypothesized that this disordered region of GraT could be the preferential binding site for DnaK. To test this possibility, we analyzed whether *N*-terminally truncated GraT lacking the first 22 residues (Δ22GraT) can co-purify with His_6_-DnaK. The pull-down assay shows that Δ22GraT can interact with DnaK (Figure 4), indicating that the disordered *N*-terminus of the toxin is not essential for DnaK binding.

### 3.5. The Negatively Charged Motif in the DnaK C-Terminus Contributes to Competitive Fitness of P. putida Not Only at High but Also at Optimal Temperature

**Figure 5:**
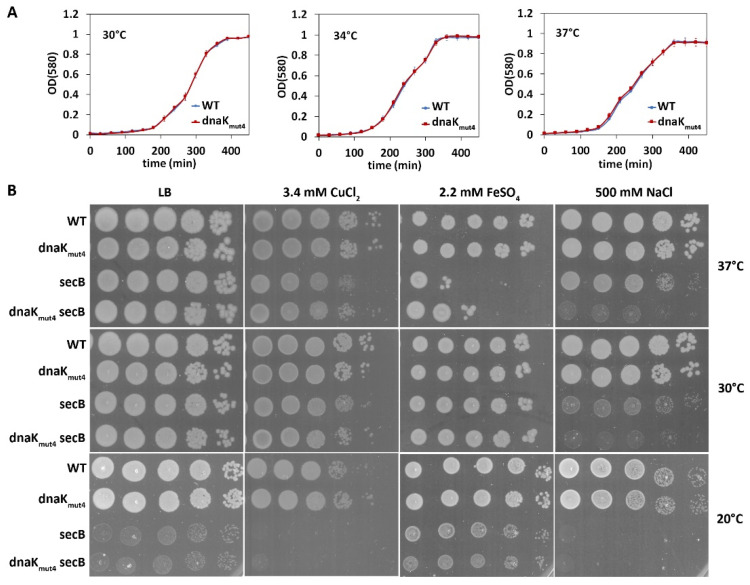
Mutation of the *C*-terminal conserved motif of DnaK results in no growth or heat tolerance defect in wild-type background, but affects the stress tolerance of *secB* deficient strain. (**A**) Growth curves of *P. putida* wild-type (WT) and *dnaK_mut4_* strains in LB medium at 30 °C, 34 °C and 37 °C. The strains were grown on microtiter plates. The results are the average of at least 15 replicates and error bars represent the standard deviation. (**B**) Solid medium stress tolerance assays of *P. putida* wild-type and *dnaK_mut4_* strains and their *secB* deficient derivatives. Tenfold serial dilutions of the overnight cultures were spotted onto LB medium and on LB supplemented with NaCl or metal salts. Final concentrations of the chemicals are indicated. Plates were incubated at indicated temperature for 24 h (LB and LB + 3.4 mM CuCl_2_ at 37 °C and 30 °C) or 48 h.

The finding that the conserved *C*-terminal motif in DnaK plays an important role in supporting GraT toxicity prompted us to study other fitness effects that the DnaK *C*-terminus might have in *P. putida*. First, the growth of the wild-type and *dnaK_mut4_* strain was compared at different temperatures. Given that growth curves of two strains revealed no clear differences in any tested temperatures (Figure 5A), the substitution mutations in the DnaK *C*-terminus seem not to significantly influence the growth and thermotolerance of *P. putida*. To control whether temperatures higher than 30 °C can trigger the heat shock response, the activation of the σ^32^-dependent P*ibpfxs* promoter was analyzed. Data show that transcription from P*ibpfxs* was upregulated already at 34 °C and even more at 38 °C (Supplementary Figure S1). Thus, despite the unaffected growth (Figure 5A), these temperatures do represent heat stress to *P. putida*.

Data in Figure 2C indicate that the streptomycin tolerance of the *dnaK_mut4_* strain is slightly increased. This prompted us to compare the wild-type and *dnaK_mut4_* strains under stress conditions that are known to boost protein misfolding and aggregation. Analysis of metal and sodium chloride tolerance revealed no clear difference between the wild-type and *dnaK_mut4_* strain at any tested temperature (Figure 5B). However, as defects in DnaK can be masked by other cellular chaperones [7,52], we also analyzed the temperature and stress tolerance of the *dnaK_mut4_* derivative deficient in chaperone SecB. Our data show that, similar to *E. coli* [52], deficiency in *secB* results in a cold-sensitive growth defect in *P. putida* (Figure 5B). Comparison of the *dnaK_mut4_ secB* double mutant to the *secB* strain revealed that the double mutant was remarkably more sensitive to NaCl stress at 37 °C and 30 °C (Figure 5B). Contrary to that, however, the *dnaK_mut4_ secB* strain was less sensitive to the excess of iron and also slightly to copper at 37 °C than the *secB* strain (Figure 5B). This indicates that the negatively charged motif in the DnaK *C*-terminus contributes to DnaK functionality, but its effects can be either positive or negative and seem to depend on the stress condition.

**Figure 6:**
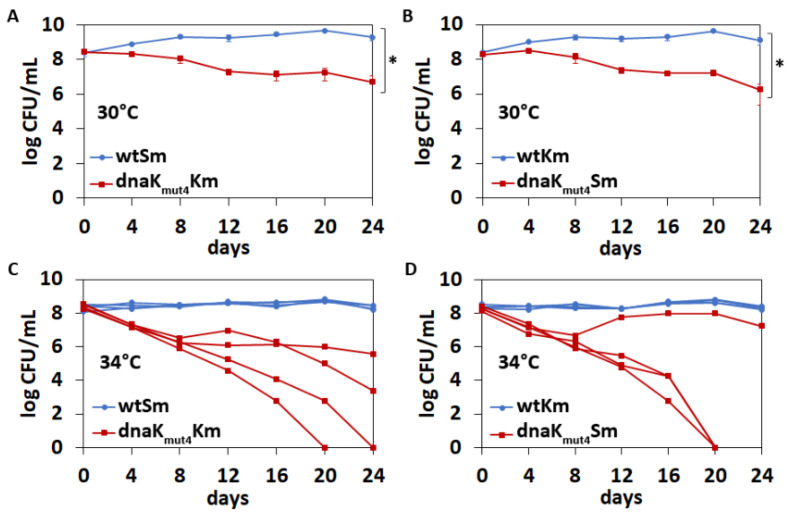
The negatively charged conserved motif in DnaK *C*-terminus is needed for competitive fitness of *P. putida*. Co-cultivation of *P. putida* wild-type and *dnaK_mut4_* strains that are marked with an antibiotic resistance gene (streptomycin or kanamycin). Panels A and C represent data for wtSm:*dnaK_mut4_*Km cocultivation mixtures and panels B and D for wtKm:*dnaK_mut4_*Sm mixtures. Bacteria were grown in LB medium at 30 °C or at 34 °C, diluted into a fresh LB medium every 2 days and CFU/mL was measured every 4 days. Means of four independent co-cultures with standard deviations are presented for experiments at 30 °C (**A**,**B**). For data obtained at 34 °C (**C**,**D**), each independent co-culture is graphed. Two-way ANOVA test was used to evaluate the difference between two strains over all time points (* *p* < 10^−16^).

In order to test whether the DnaK *C*-terminal end can influence the competitive fitness of *P. putida*, a co-culture competition assay with mixed populations of wild-type and *dnaK_mut4_* strains was performed. Both strains were marked with kanamycin and streptomycin resistance genes. Growth curve analysis revealed that resistance genes had no effect on bacterial growth (Supplementary Figure S2). For the competition assay, 1:1 mixtures of wtKm:*dnaK_mut4_*Sm as well as wtSm:*dnaK_mut4_*Km were grown during 24 days in LB medium at 30 °C and 34 °C. Experiments at 37 °C were not possible as *P. putida* could not tolerate long-term cultivation at higher temperatures and bacteria died after the fourth day of co-cultivation. Over the 24 days of competition, bacteria pass about 144 generations, which is about ten times more than during growth curve experiments. The competition assay shows that the *C*-terminal conserved motif is important for DnaK functionality already at optimal temperature, as the *P. putida* wild-type strain displayed significant competitive advantage over the *dnaK_mut4_* strain at 30 °C (Figure 6A,B). Co-cultivation at 34 °C revealed a highly important role of the DnaK *C*-terminus for long-term thermotolerance of *P. putida* because the *dnaK_mut4_* strain was outcompeted from most mixed cultures of wild-type and *dnaK_mut4_* (Figure 6C,D). Interestingly, the out-competition dynamics of the *dnaK_mut4_* strain in separate mixed populations varied greatly at 34 °C, particularly after the 8th day of experiment, and there were some cultures where the ratio of *dnaK_mut4_* strain in the mix started to increase during the experiment (Figure 6C,D). The different fate of the *dnaK_mut4_* strain in parallel co-cultures suggests that the *dnaK_mut4_* strain experiences a high selection pressure, which may lead to regulatory or mutational changes.

## 4. Discussion

TA systems are potentially deleterious to their host bacterium, and it is therefore not surprising that TA modules are tightly controlled. Most control over the toxin activity occurs through the regulation of antitoxin expression and stability. Besides the well-documented fact that the stability of antitoxins is affected by cellular proteases [32], there are also reports of protein folding chaperones being involved in correct folding and thus the stabilization of antitoxins [15,16]. Curiously, the current study demonstrates that toxin activity can also be positively affected by a cellular chaperone, as we show that DnaK facilitates the toxicity of GraT in the absence of its cognate antitoxin GraA. Another interesting finding of this study is that the enigmatic *C*-terminal disordered end of DnaK is important in shaping GraT toxicity. Furthermore, the conserved negatively charged motif in the DnaK *C*-terminus is necessary for *P. putida* competitive fitness.

The recruitment of a major host chaperone as a positive control factor of a toxin’s activity is highly surprising, particularly when considering that GraT can fully arrest the host growth. The most reasonable explanation for chaperone addiction of GraT is that this toxin is intrinsically unable to fold correctly. This is supported by structural analysis of the GraTA complex, which has revealed that the 22 amino acid-long *N*-terminus of GraT is disordered [29]. Interestingly, the disordered region of GraT has an important role in the regulation of the *graTA* operon, as this flexible segment hinders the binding of the GraTA complex to the operator and thus leads to the derepression of *graTA* transcription [29]. The switch between the GraA-mediated repression and the GraTA complex-mediated derepression of the *graTA* operon is highly relevant under conditions of high toxin:antitoxin ratio, as this allows de novo synthesis of antitoxin and thus prevents the accidental poisoning of host bacterium by the toxin GraT.

The unstructured *N*-terminus of GraT not only regulates *graTA* expression, but is also important for GraT toxicity and mRNase activity [29]. Structural data predict that the catalytically active site for mRNA degradation is located in the distal *C*-terminus of GraT, and that the *N*-terminus does not contribute to catalysis [29]. However, GraT cleaves mRNAs in a ribosome-dependent manner and should bind to the ribosome. Unfortunately, despite several efforts, we have not succeeded in isolating the ribosome-GraT complexes, indicating that the interaction between GraT and ribosome is weak and easily disrupted during ribosome purification. We propose that the *N-*terminal disordered segment of GraT could complicate the stable binding of the toxin to the ribosome, and this is probably the reason why the assistance of DnaK is required. Supporting evidence for the involvement of the toxin *N*-terminus in ribosome binding can be found from the structural analysis of the HigB of *P. vulgaris* [53]. *P. vulgaris* HigB is the closest homologue of GraT, and, except for the *N-*terminal disordered part, the structure of GraT resembles that of HigB [29,34]. Structural analysis of ribosome-HigB complexes showed that *P. vulgaris* HigB binds to the ribosomal A-site through two solvent-exposed basic regions [54], and, intriguingly, one of those interaction surfaces is located in the HigB *N*-terminus, i.e., just in the region which is unstructured in GraT. Substitutional mutagenesis of the HigB *N*-terminus suppressed its toxicity, which was considered to result from the decreased binding of HigB to the ribosome [54]. Analogously, the *N-*terminal truncation of GraT has been shown to eliminate the toxin mRNase activity and toxicity to the host [29]. We assume that, similar to HigB, the *N*-terminus of GraT participates in ribosome binding, but as it is intrinsically disordered, the assistance of DnaK is needed to fold GraT into the ribosome-binding-competent conformation.

An interesting feature of GraT toxin is that it causes cold-sensitive phenotypes. GraT greatly suppresses the growth of the antitoxin deletion strain *ΔgraA* at 20 °C, but only slightly decreases the growth rate at 30 °C and has no effect on growth at 37 °C [30]. The involvement of the DnaK chaperone as a positive factor in GraT functionality suggests a plausible explanation for the temperature-sensitivity of GraT toxin. Higher temperatures cause the accumulation of misfolded and aggregated proteins and DnaK has a central role in their refolding and disaggregation [5]. It is therefore reasonable to assume that the interaction of DnaK with damaged proteins during heat stress titrates the chaperone away from binding to GraT. Hence, the deprivation of GraT of folding assistance at elevated temperatures results in non-toxic GraT. At lower temperatures, more DnaK is available for GraT binding and folding, and this results in more severe effects of GraT on cell growth. An alternative, but not mutually exclusive, explanation is that the spontaneous folding of GraT depends on temperature and occurs more correctly at lower temperatures.

Extensive studies have provided a detailed picture of the DnaK molecular mechanism that is regulated by the allosteric interactions between its nucleotide-binding domain and substrate binding domain [6]. However, much less is known about the biological role of the distal *C*-terminal disordered domain, which is not essential for DnaK chaperone activity [13,14]. To the best of our knowledge, the functional significance of the *C*-terminal domain of a bacterial DnaK is addressed in only two studies, which inconsistently propose that the extreme *C*-terminus may either reduce [8] or enhance the protein refolding activity of DnaK [7]. Our data are more consistent with the latter possibility because the *C*-terminal end positively impacts on DnaK’s ability to enhance GraT toxicity. Mutagenesis of the *C*-terminal conserved negatively charged motif ^625^DDVVDAEFEEVKD^637^ shows that this motif has a major role in the DnaK-mediated enhancement of GraT toxicity (Figure 2). The other clear evidence for the positive effect of the *C*-terminal charged motif to DnaK functionality came from the competition assay, as replacing the charged ^629^DAEFEE^634^ motif in the extreme end of DnaK with the sequence ^629^NAQFQQ^634^ resulted in decreased competitive fitness of *P. putida* at optimal temperature, and even more at a higher temperature (Figure 6). We consider these results especially interesting because this is the first time when the mutagenesis of the DnaK *C*-terminal domain has resulted in a measurable deficiency of bacteria that have an otherwise wild-type genotype. In *E. coli*, DnaK *C*-terminal truncation has been shown to cause growth defects upon heat shock or SecB depletion [7]. However, these data were obtained with the *E. coli* derivative that has, in addition to *dnaK C*-terminal deletion, reduced levels of *dnaJ* expression as well as harbored a sigma S mutation that results in lower heat shock response [7,55]. Given that other cellular chaperones and heat shock proteins can compensate for the DnaK deficiency [7,56], such genetic background allowed the appearance of phenotypes caused by the *C*-terminal truncation of DnaK. Similar compensation of a *C*-terminal defect of DnaK seems to also occur in *P. putida*, as the growth and stress tolerance of the *dnaK_mut4_* strain were mostly equal to wild-type (the only exception was the slightly increased streptomycin tolerance of *dnaK_mut4_* compared to the wild-type; Figure 2C), and only when *secB* was disrupted did the defect of the *dnaK_mut4_* become evident in some stress conditions (Figure 5). However, the effect of the mutated DnaK_mut4_ in the *secB* strain depended on the stress condition applied. Compared to the parent *secB* strain, the *secBdnaK_mut4_* derivative was less tolerant to NaCl stress but more tolerant to the excess of iron and copper (Figure 5). These data suggest that the *C*-terminal end of DnaK can have both positive and negative fitness consequences, at least when the chaperone SecB is deficient. Still, considering that the mutagenesis of the *C*-terminal motif in DnaK essentially decreased the long-term thermotolerance and competitive fitness in wild-type *secB* background, the disordered *C*-terminal domain confers a clear fitness advantage and therefore enhances rather than decreases the chaperone activity of DnaK.

It has been proposed that the unstructured *C*-terminus of DnaK comprises an auxiliary binding site for denatured client proteins, and that this additional transient binding would allow it to keep the client protein close to the chaperone after it is released from the canonical substrate-binding site [7]. This may support the repeated folding cycles of DnaK, and thus, facilitate the processing of misfolded proteins. Considering that many eukaryotic homologs of DnaK, the Hsp70s, also possess intrinsically disordered *C*-termini which contribute to substrate recognition and co-chaperone binding [57], the functioning of the *C*-terminal structural disorder in protein recognition seems to be evolutionarily conserved across the bacterial and eukaryotic chaperones.

## Figures and Tables

**Table 1:** Bacterial strains and plasmids.

Strain or Plasmid	Genotype or Characteristic(s)	Source or Reference
*E. coli* strains		
DH5α λ*pir*	λ*pir* lysogen of DH5α	[41]
BL21(DE3)	*hsdS gal* (λ*c*I *ts*857 *ind-1 S*am7 *nin-5 lacUV5*-T7 gene *1*)	[42]
*P. putida* strains		
PaW85	Wild-type, isogenic to KT2440	[37]
∆*graA*	PaW85 ∆*graA*	[30]
tac-*dnaK*	PaW85 with *lacI*^q^-*P*_tac_*-dnaK* expression cassette in *glmS* locus (Sm^r^)	This study
∆*graA* tac-*dnaK*	∆*graA* with *lacI*^q^-*P*_tac_*-dnaK* expression cassette in *glmS* locus (Sm^r^)	This study
∆*graA dnaK_Δ41_*	∆*graA* with 41 *C*-terminal amino acids truncated from DnaK	[17]
*dnaK_mut4_*	DnaK carries substitution mutations D629N, E631Q, E633Q and E634Q	This study
∆*graA dnaK_mut4_*	∆*graA* with DnaK carrying mutations D629N, E631Q, E633Q and E634Q	This study
*secB*	PaW85 *secB*::Sm (Sm^r^)	This study
*dnaK_mut4_ secB*	*dnaK_mut4_* with *secB*::Sm (Sm^r^)	This study
wtSm	PaW85 with miniTn7-ΩSm in *glmS* locus	[38]
wtKm	PaW85 with miniTn7-Km in *glmS* locus	[38]
*dnaK_mut4_*Sm	*dnaK_mut4_* with miniTn7-ΩSm in *glmS* locus	This study
*dnaK_mut4_*Km	*dnaK_mut4_* with miniTn7-Km in *glmS* locus	This study
Plasmids		
pUCNot-lacItac	pUC18Not with *lacI*^q^-*P*_tac_ cassette (Amp^r^)	This study
pUCNotlacItac-dnaK	pUC18Not with *lacI*^q^-*P*_tac_*-dnaK* expression cassette (Amp^r^)	This study
pBK-miniTn7-ΩSm	pUC19-based delivery plasmid for miniTn7-ΩSm (Amp^r^ Sm^r^)	[43]
pminiTn7lacItac-dnaK	pBK-miniTn7-ΩSm with *lacI*^q^-*P*_tac_*-dnaK* expression cassette (Amp^r^ Sm^r^)	This study
pBK-miniTn7-Km	pUC19-based delivery plasmid for miniTn7-Km (Amp^r^ Km^r^)	[38]
pUXBF13	Plasmid coding for the Tn7 transposition proteins (Amp^r^ *mob*^+^)	[44]
pEMG	Suicide plasmid containing *lacZ*α with two flanking I-SceI sites (Km^r^)	[41]
pSW(I-SceI)	Plasmid for I-SceI expression (Ap^r^)	[45]
pEMG-dnaK_mut4_	pEMG with a PCR-designed 1040 bp XbaI-KpnI insert containing substitution mutations D629N, E631Q, E633Q and E634Q in the DnaK *C*-terminus	This study
pET11c	Protein expression vector (Ap^r^)	Lab collection
pET-hisDnaK	pET11c for expression of DnaK with *N*-terminal His_6_ tag (Ap^r^)	[17]
pET-hisDnaK_C57_	pET11c for expression of His_6_-DnaK_C57_, 57 *C*-terminal amino acids of DnaK are fused with *N*-terminal His_6_ tag (Ap^r^)	This study
pET-graT_Δ1C_	pET11c for expression of GraT_Δ1C_ (Ap^r^)	[17]
pET-Δ22graT	pET11c for expression of *N*-terminally truncated Δ22GraT (Ap^r^)	[29]
pT25	Plasmid encoding the T25 fragment (1–224 amino acids) of *cyaA* (Km^r^)	[46]
pT18	Plasmid encoding the T18 fragment (225–399 amino acids) of *cyaA* (Ap^r^)	[46]
pT25-zip	Plasmid encoding the T25 fragment fused with leucine zipper (Km^r^)	[46]
pT18-zip	Plasmid encoding the T18 fragment fused with leucine zipper (Ap^r^)	[46]
pT25-graT	Plasmid encoding the T25 fragment fused with *graT* (Km^r^)	This study
pT18-graA	Plasmid encoding the T18 fragment fused with *graA* (Ap^r^)	This study
pT18-dnaK_C44_	Plasmid encoding the T18 fragment fused with 44 *C*-terminal amino acids of DnaK (Ap^r^)	This study
pKS-secB	pBluescript KS containing PCR-amplified *secB* (PP_5053) (Amp^r^)	This study
pUTmini-Tn5Sm/Sp	Delivery plasmid for mini-Tn5Sm/Sp (Amp^r^ Sm^r^)	[47]
pKS-secB::Sm	Central 280 bp region of *secB* in pKS-secB is replaced by Sm^r^ gene (Amp^r^ Sm^r^)	This study
pGP704L	Delivery plasmid for homologous recombination (Amp^r^)	[48]
p704L-secB::Sm	pGP704L with Acc65I-SacI fragment of secB::Sm from pKS-secB::Sm (Amp^r^ Sm^r^)	This study
pRK2013	Helper plasmid for conjugal transfer of pGP704L	[49]
pAG032	Broad-host-range reporter plasmid carrying P*ibpfxs-cfp*, P*rpsJ-yfp* and Pm-*rfp* promoter/reporter pairs	[50]

**Table 2:** Oligonucleotides.

Name	Sequence (5’-3’) *^a^*	Use
DnaKBam	aaggatccaaagtagtcgctgctacc	construction of pUCNotlacItac-dnaK
dnaJdel	aatcacgcttggacataggt	construction of pUCNotlacItac-dnaK and pT18-dnaK_C44_
dnaKKpn	ccggtaccattcacgtgctgcaagg	construction of pEMG-dnaK_mut4_
dnaK-3E1D	tgtctttcacttGttGgaactGggcgtTaaccacgtcat	construction of pEMG-dnaK_mut4_
K-3E	CagttcCaaCaagtgaaagacaacaacaag	construction of pEMG-dnaK_mut4_
dnaJXba	atgcctgcaggtcgactctagatcgacacctgcatggccatact	construction of pEMG-dnaK_mut4_
secBalg	ctgctcgagagcaagggcgt	construction of pKS-secB
secBlopp	ggctctagaacgggtttgccgggag	construction of pKS-secB
T25-graT	aaactgcagcgattcgaagctttagctgtgc	construction of pT25-graT
1586Bam	cgggatccgttcttgagcatgatgc	construction of pT25-graT
T18-graA	aaagtcgacgctcaagaacggtatgcgtc	construction of pT18-graA
1585Bam	atggatccgtttttcgatgtcagtc	construction of pT18-graA
T18-dnaKsaba	tctggtaccggttgcccagaagatgta	construction of pT18-dnaK_C44_
his-dnaK(C57)	aatcatatgcatcaccaccaccatcacgacgccaaggttgaagagct	construction of pET-hisDnaK_C57_
dnaKSmaBam	attggatcccccgggattactgcttgttgttg	construction of pET-hisDnaK_C57_
dnaKqkeskFw	gtgccgaagtcctgaagaaa	qRT-PCR
dnaKqkeskRev	ctggctgtcgttgaagtagg	qRT-PCR
rpoDqFw	gcaacagcagtctcgtatca	qRT-PCR
rpoDqRev	atgatgtcttccacctgttcc	qRT-PCR

*^a^* The sites of restriction enzymes used in cloning are underlined. The mutated nucleotides are in uppercase letters.

## Data Availability

Data are contained within the article.

## Supplementary Materials

The following are available online at https://www.mdpi.com/2076-2607/9/2/375/s1, Figure S1: Heat shock response of *P. putida*, Figure S2: Growth curves of *P. putida* wild-type and *dnaK_mut4_* strains possessing streptomycin or kanamycin resistance genes.
