# Supplementary material for: The Disordered C-Terminus of the Chaperone DnaK Increases the Competitive Fitness of Pseudomonas putida and Facilitates the Toxicity of GraT
Source: Microorganisms. 2021 Feb 13;9(2):375. doi: 10.3390/microorganisms9020375 (PMC7918210; doi:10.3390/microorganisms9020375)
Supplement: Supplementary file 1 [file microorganisms-09-00375-s001.pdf]

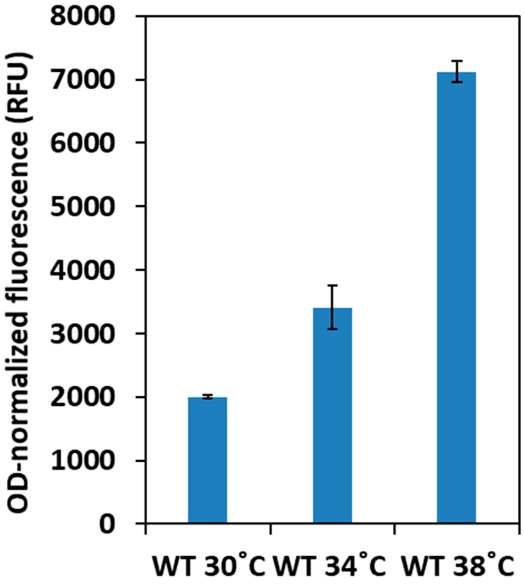

Figure S1. Heat shock response of *P. putida* measured as fluorescence of CFP (cyan fluorescent protein) expressed from the σ^{32}-dependent P*ibpfxs* promoter. *P. putida* harbouring the reporter plasmid pAG032 was grown at 30 °C in glucose minimal medium until OD_{600}~0.5. Then, some cultures were shifted to either 34 °C or 38 °C and after 2 hours of the temperature shift the OD_{600} and fluorescence (excitation 433 nm, emission 475 nm) values were measured. The OD_{600}-normalized fluorescence values (RFU) from three independent biological replicates (average and standard deviation) is presented.

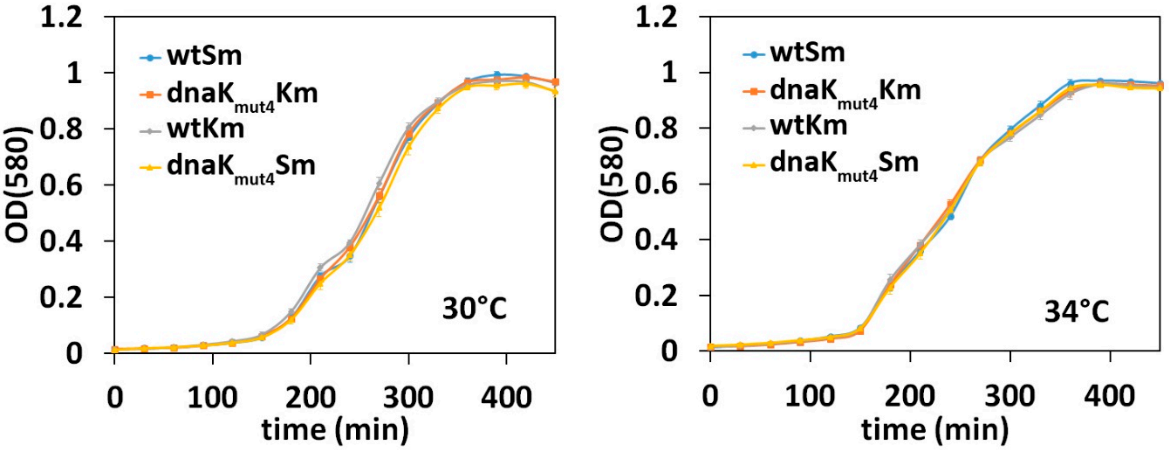

Figure S2. Growth curves of *P. putida* wild-type and $dnaK_{mut4}$ strains possessing streptomycin (wtSm and $dnaK_{mut4}$Sm) or kanamycin (wtKm and $dnaK_{mut4}$SKm) resistance genes in their chromosome. Bacteria were grown on microtiter plates in LB medium at 30 ˚C and 34 ˚C. The results are the average of at least 10 replicates and error bars represent the standard deviation.
